# Draft Genome Sequences of Two Acidophilic, Mesophilic Verrucomicrobial Methanotrophs Contain Only One *pmoCAB* Operon

**DOI:** 10.1128/MRA.00315-20

**Published:** 2020-04-16

**Authors:** Geert Cremers, Arjan Pol, Mike S. M. Jetten, Huub J. M. Op den Camp

**Affiliations:** a Department of Microbiology, IWWR, Radboud University, Nijmegen, The Netherlands; Broad Institute

## Abstract

Methylacidimicrobium cyclopophantes 3B and Methylacidimicrobium tartarophylax 4AC are Gram-negative rod-shaped mesophilic methanotrophs isolated from soil samples with low pH at the Solfatara Crater, near Naples, Italy. The genomes of these extremophilic verrucomicrobia were sequenced using Illumina technology, and both species possess one *pmoCAB* operon and two *xoxF* genes.

## ANNOUNCEMENT

Methane is a potent greenhouse gas, and methanotrophs play a significant role in mitigating methane emissions to the atmosphere. Methane-oxidizing bacteria have been detected in a variety of environments, including the hot and acidic soils of volcanic regions (1). The majority of the methanotrophs in these hostile environments belong to the verrucomicrobial methanotrophs. *Methylacidiphilum* species growing at 40°C to 60°C and pH 1 to 6 were described in 2007 (2). Later, mesophilic, highly acid-tolerant isolates belonging to a second genus, *Methylacidimicrobium*, were discovered (3, 4). Here, we report the draft genome sequences of two representatives of this genus (3). Methylacidimicrobium cyclopophantes 3B and Methylacidimicrobium tartarophylax 4AC were isolated from soil samples taken at the Solfatara Crater, at the center of the Campi Flegrei caldera, near Naples, Italy, and were phylogenetically characterized (3). The strains were grown in a medium containing lanthanides (5) based on local soil concentrations and with a headspace of 5% (vol/vol) carbon dioxide and 10% (vol/vol) methane at 29°C with shaking at 350 rpm.

DNA was isolated using the DNeasy Powersoil kit (Qiagen, Venlo, The Netherlands) with 5-min sonication at 15-s intervals (Bioruptor Next Gen; Diagenode AS, Ougrée, Belgium), and a library was prepared according to the manufacturer’s protocol (Nextera DNA sample preparation kit; Illumina, San Diego, CA, USA). The library was single-end sequenced using the cBot single-read cluster generation system (catalog number GD-300-1001) and a 36-cycle sequencing kit V4 (catalog number FC-104-4002) on the Genome Analyzer II system (Illumina). The quality of the sequence reads was checked with CLC workbench 4 (Qiagen Aarhus A/S, Denmark) and trimmed using a quality limit of 0.001 and minimum number of nucleotides in reads of 30. Assemblies were performed using CLC workbench 4 (Qiagen Aarhus A/S) with default settings and a minimum contig length of 500 bp. Binning was done on G+C content (no threshold) and coverage (>250×) by plotting G+C content against coverage per contig in a scatterplot. For *Methylacidimicrobium tartarophylax* 4AC, the trimmed reads (27,600,863) resulted in 137 contigs ranging from 502 to 140,500 bp (*N*_50_, 31,024 bp). For *Methylacidimicrobium cyclopophantes* 3B, the trimmed reads (29,990,414) resulted in 273 contigs ranging from 513 to 63,014 bp (*N*_50_, 14,841 bp). Completeness (strain 3B, 98.7%; strain, 4AC, 98.0%) and contamination (strain 3B, 0.7%; strain 4AC, 1.4%) were determined using CheckM (6). The genomes were annotated by the NCBI Prokaryotic Genome Annotation Pipeline (PGAP) (7) and analyzed with emphasis on the major methanotrophic pathways. Additional information on the genome sequences is compiled in Table 1.

**TABLE 1:** Characteristics of the *Methylacidimicrobium* genomes

Isolate	Genome size (bp)	G+C content (%)	No. of contigs	*N*_50_ (bp)	Coverage (×)	No. of genes by type				No. of CRISPR arrays
						Total	CDS^*a*^	RNA	Pseudogenes	
*M. tartarophylax* 4AC	2,327,085	61.2	137	31,024	665	2,241	2,188	53	121	1
*M. cyclopophantes* 3B	2,276,790	61.2	273	14,841	598	2,178	2,121	57	141	3

a CDS, coding DNA sequences.

Unlike the *Methylacidiphilum* species (8–10) which contain three *pmoCAB* operons, strains 3B and 4AC possess only one *pmoCAB* operon encoding the membrane-bound methane monooxygenase. Furthermore, they also lack genes encoding the soluble methane monooxygenase (*mmoXYZ*). Both genomes contain two *xoxF* genes encoding lanthanide-dependent pyrroloquinoline quinone (PQQ)-methanol dehydrogenases (5, 11), the accompanying *xoxG* (encoding the electron acceptor *cytochrome c*) and *xoxJ* genes, and all genes for cofactor PQQ synthesis. All genes required for complete oxidation of formaldehyde/formate were identified. In addition, the genomes encode a complete Calvin cycle for CO_2_ fixation, including the genes for two subunits of the key enzyme RuBisCO (*cbbL* and *cbbS*) (3, 12). The presence of hydrogenase gene clusters in both genomes supports potential growth as chemolithotrophic Knallgas bacteria (13, 14). Both strains contain *nifHDK* genes, indicating that, similar to Methylacidiphilum fumariolicum SolV, nitrogen fixation might be possible (15).

### Data availability.

This whole-genome shotgun project has been deposited in ENA under project number PRJEB32513. The assembled genomes are deposited under accession numbers GCA_902143385 and GCA_902143375. The versions described in this paper are the second versions. Raw reads are available under SRA accession numbers ERR3674889 and ERR3675335.
